# Circumferential Skin and Subcutaneous Tissue Necrosis of the Left Lower Leg Secondary to a Chronic Arterial Ulcer Complicated by Bullous Cellulitis: Staged Debridement, NPWT, and Split-Thickness Skin Grafting for Limb Salvage

**DOI:** 10.7759/cureus.103184

**Published:** 2026-02-07

**Authors:** Ivan Dilber

**Affiliations:** 1 Plastic and Reconstructive Surgery, Zabok General Hospital, Zabok, HRV

**Keywords:** arterial ulcer, bullous cellulitis, circumferential lower-leg defect, limb salvage, negative pressure wound therapy, split-thickness skin graft

## Abstract

Chronic arterial ulcers are associated with impaired healing and a high risk of infectious complications and limb loss. Bullous cellulitis represents a severe soft-tissue infection phenotype that may progress to extensive tissue necrosis. We report an 83-year-old woman with advanced atherosclerotic disease and a prior contralateral transfemoral amputation who developed bullous cellulitis complicating a long-standing circumferential distal lower-leg arterial ulcer, resulting in complete circumferential loss of skin and subcutaneous tissue with exposure of deep structures. After infection stabilization, the wound was managed with staged mechanical and chemical debridement, negative pressure wound therapy (NPWT) for wound-bed optimization, and definitive split-thickness skin grafting under NPWT bolster. Complete graft take was achieved without early complications. Limb salvage preserved the patient’s mobility and quality of life, avoiding the functional consequences of bilateral major limb loss. The key novelty of this case is that even circumferential ischemic-infective necrosis, often presumed to mandate amputation, can be rendered graftable and salvaged through a strictly staged, NPWT-centered reconstructive strategy.

## Introduction

Circumferential skin necrosis of the lower extremity represents a rare but severe surgical challenge, carrying a substantial risk of limb loss. Patients with advanced peripheral arterial disease are particularly vulnerable to chronic ulceration due to impaired tissue perfusion, which predisposes them to delayed healing and secondary infection. Superimposed soft tissue infections, such as bullous cellulitis, may precipitate rapid progression to extensive skin and subcutaneous tissue necrosis, occasionally resulting in circumferential defects.

Arterial ulcers typically occur in the distal lower leg and are associated with significant comorbidities, including atherosclerotic disease, hypertension, hyperlipidemia, and limb loss. Previous vascular interventions or major limb amputations further reflect advanced disease and markedly increase the risk of contralateral limb complications. Management of such complex wounds requires a staged approach aimed at infection control, removal of non-viable tissue, optimization of the wound bed, and delayed reconstruction. 

This report highlights that early aggressive debridement combined with prolonged negative pressure wound therapy can convert an apparently non-salvageable circumferential ischemic-infective defect into a graftable wound, thereby avoiding amputation [1-7].

## Case presentation

An 83-year-old woman was admitted with an acute exacerbation of a chronic ulcer of the left lower leg. Her medical history was significant for advanced atherosclerotic arterial disease with chronic ulceration, hyperlipidemia, arterial hypertension, and chronic gastritis. Also, a right transfemoral amputation was performed in 2012 due to gangrene. She was a former smoker. Long-term medication included acetylsalicylic acid 100 mg daily (Andol 100), lisinopril/hydrochlorothiazide (Iruzid 20/12.5 mg), an HMG-CoA reductase inhibitor (Coupet 10 mg), esomeprazole (Emanera 40 mg), zolpidem (Lunata 10 mg), and ibuprofen (Ibuprofen Belupo 600 mg) as needed.

The patient reported an ulcer present since 2021 without prior major inflammatory exacerbations. Two days before admission, she developed progressive erythema of the surrounding skin, bullae formation, and fever. Pain was mild and not a leading symptom.

On admission, she was hemodynamically stable (blood pressure 110/70 mmHg), oriented, and afebrile under antipyretic therapy (36.1°C). Local examination revealed a distal-third lower-leg ulcer involving almost the entire circumference, with surrounding cellulitis and bullous lesions extending proximally.

Laboratory investigations showed C-reactive protein (CRP) 206.9 mg/L (reference range <0.3 mg/L) on admission, leukocytes 7.1×10⁹/L (reference range 3.4 - 9.7×10⁹/L) with relative neutrophilia. A wound swab demonstrated mixed skin flora without a dominant pathogen. Intravenous clindamycin (600 mg three times daily) and metronidazole (500 mg three times daily) were initiated.

In subsequent days, cellulitis progressed to involve the entire circumference up to the proximal third; however, with antibiotic therapy and conservative dressings, further progression ceased and inflammatory markers declined (CRP to ~50 mg/L by 22 July, ref. range <0.3mg/L). The wound then remained clinically stationary, enabling staged surgical management.

Surgical management

**Figure 1:**
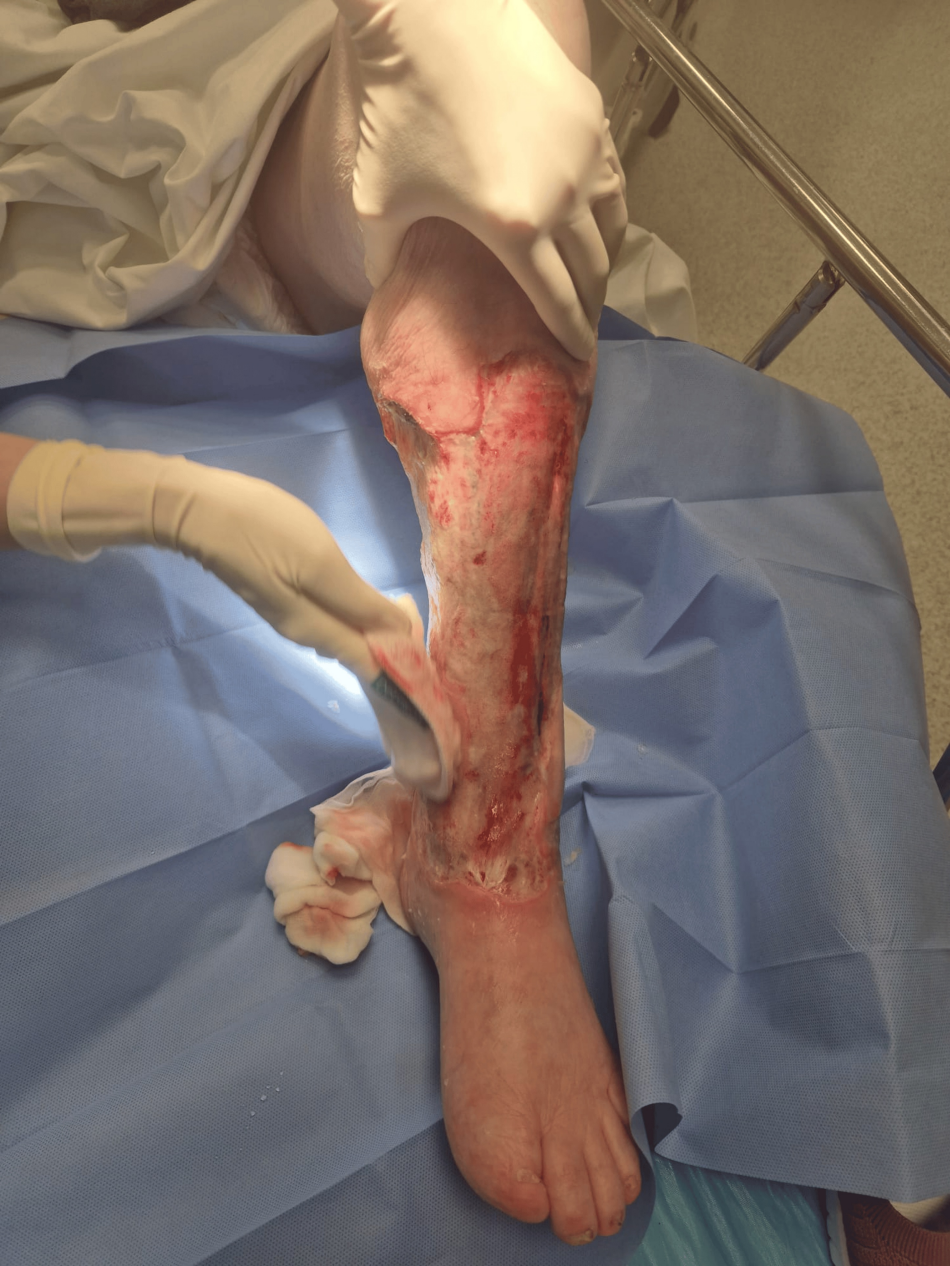
Circumferential defect of the left lower leg prior to NPWT. Aftermath of chronic distal lower-leg ulcer complicated by bullous cellulitis with circumferential skin/subcutaneous tissue loss and exposure of deep structures (deep fascia and underlying tendons). NPWT: Negative Pressure Wound Therapy

Given circumferential tissue loss, exposure of deep structures, and the substantial risk that recurrent infection could necessitate a second major amputation, a limb-salvage strategy was pursued (Figure 1).

Three serial mechanical debridements were performed using scalpel excision, complemented by chemical debridement (Granugel) between procedures. Partial fasciotomies were undertaken in areas of necrotic involvement. By the time of definitive wound-bed preparation, overt necrosis was no longer present following serial surgical debridement; the defect demonstrated circumferential exposure of fascia and muscle with extensive fibrinous deposits. Subsequent microbiological wound swabs were negative, and there were no clinical or laboratory signs of ongoing infection.

**Figure 2:**
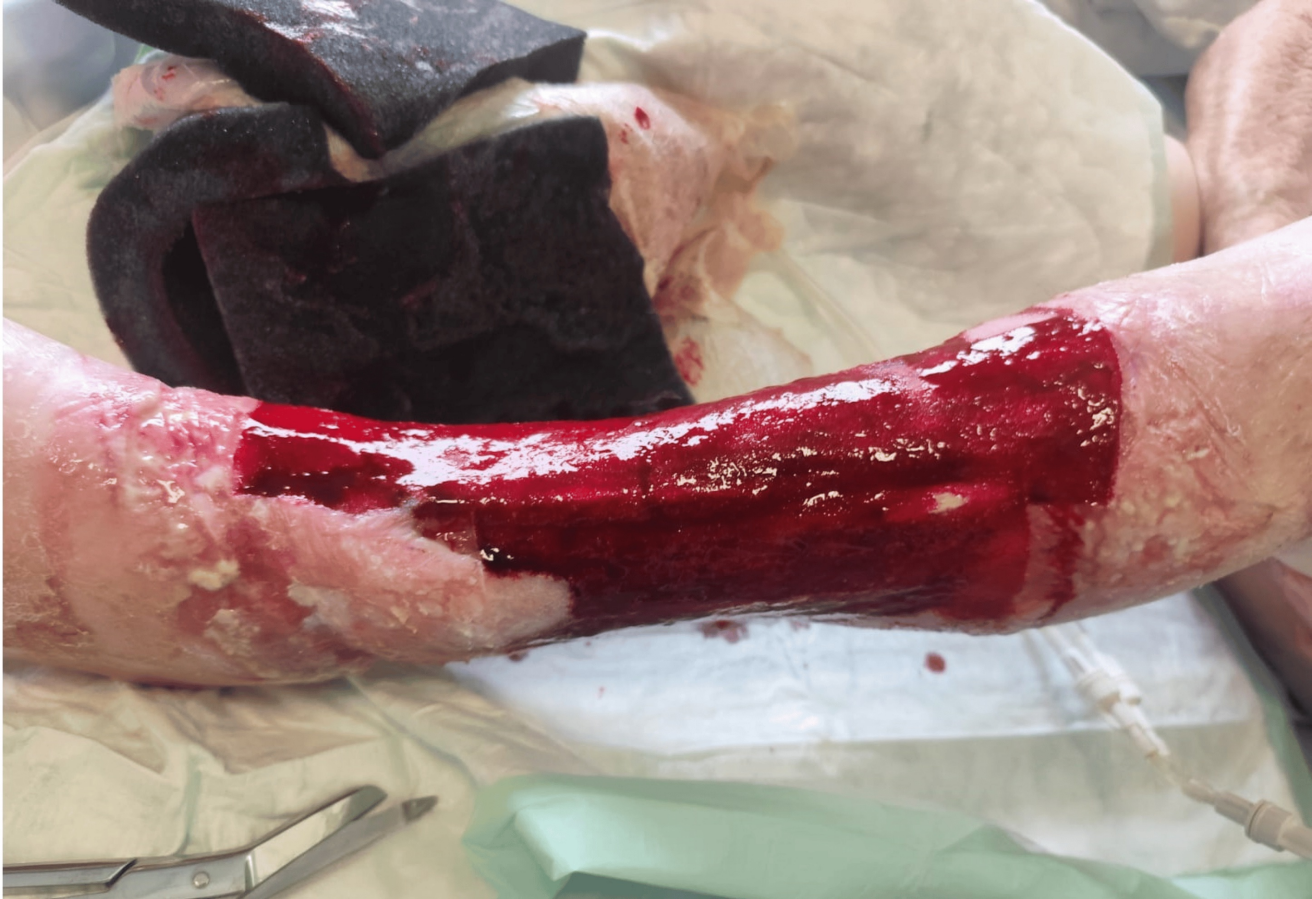
Wound appearance after serial debridement and NPWT. Clean, granulating wound bed without residual necrosis, suitable for definitive coverage.

A final radical debridement was performed, consisting of sharp excision of devitalized tissue and mechanical cleansing using a sterile brush. NPWT was applied from POD-1 to POD-7, achieving a clean, granulating wound bed suitable for coverage (Figure 2).

**Figure 3:**
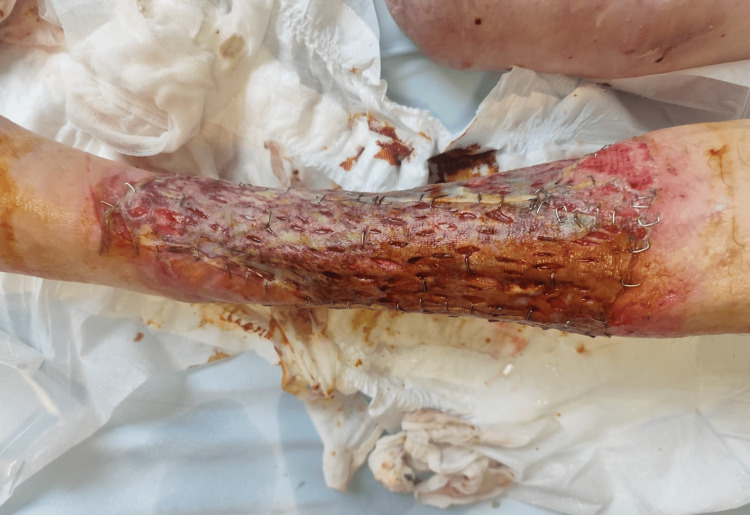
Immediate postoperative appearance after split-thickness skin grafting. Complete circumferential coverage prior to NPWT bolster application.

Definitive reconstruction was performed on 29 July with a split-thickness skin graft harvested from the ipsilateral leg on the front side of the thigh, measuring three grafts sized 20 cm x 5 cm; no dermal substitute was used. NPWT was reapplied as a graft bolster for ten days. The immediate postoperative graft appearance is shown in Figure 3.

Outcome and follow-up

The postoperative course was uncomplicated. Complete graft adherence was achieved with no evidence of infection, hematoma, seroma, or graft loss. Hospitalization lasted for 29 days.

Discharge instructions included strict limb elevation and avoidance of limb use until the first follow-up, with PHMB (polyhexamethylene biguanide) dressings every 3-4 days. Oral amoxicillin/clavulanic acid 1 g twice daily for seven days was prescribed, and acetylsalicylic acid 100 mg daily was continued along with existing chronic therapy.

**Figure 4:**
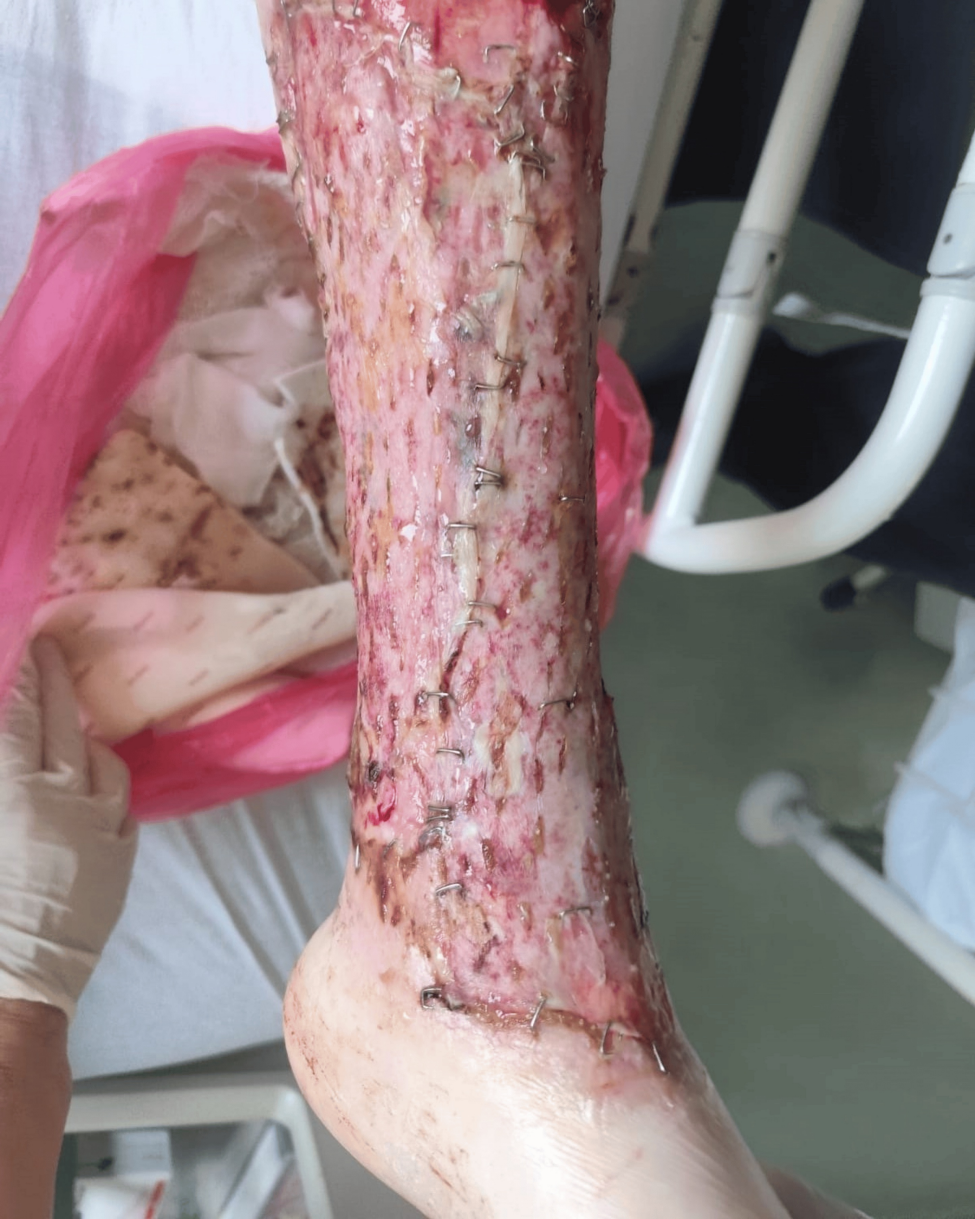
First follow-up seven days after discharge. Fully viable graft without infection or necrosis and satisfactory early healing.

At the first outpatient follow-up, seven days after discharge, the graft remained fully viable without complications (Figure 4).

Continued PHMB dressings were recommended for an additional week, together with gradual verticalization and resumption of use of the contralateral prosthesis. The patient expressed high satisfaction, noting that limb salvage preserved independence and avoided the prospect of bilateral major amputation.

## Discussion

Circumferential soft tissue necrosis of the lower limb represents an uncommon but limb-threatening condition, most frequently reported in the setting of severe infection, trauma, or critical limb ischemia [4]. In patients with advanced peripheral arterial disease, chronic ulceration creates a vulnerable substrate in which superimposed infection may rapidly progress to extensive tissue loss [4]. Bullous cellulitis, although typically managed conservatively, can in compromised hosts precipitate aggressive soft tissue destruction and circumferential necrosis, markedly increasing the risk of major amputation [4].

Previous reports addressing extensive lower-limb soft tissue necrosis emphasize early amputation or complex flap-based reconstruction, particularly when deep structures are exposed [4]. However, outcomes in ischemic and infected fields remain unpredictable, and reconstructive options are often limited by poor vascular status, patient comorbidities, and high perioperative risk. In contrast, the present case demonstrates that a strictly staged approach, centered on serial debridement, prolonged negative pressure wound therapy (NPWT), and delayed split-thickness skin grafting, can achieve limb salvage even in the presence of circumferential tissue loss [4,7].

The effectiveness of the chosen strategy can be attributed to several factors. Serial debridement allowed for gradual and accurate delineation of tissue viability while avoiding excessive initial excision that could have further compromised limb integrity. Prolonged NPWT played a pivotal role in wound bed preparation by promoting granulation tissue formation over exposed fascia and muscle, reducing edema, and controlling bacterial burden [7,8]. Importantly, repeated microbiological sampling confirmed the absence of ongoing infection prior to definitive reconstruction, in line with established principles for managing complex soft tissue infections [9,10]. Once a stable, well-vascularized wound bed was achieved, split-thickness skin grafting provided reliable coverage with minimal donor-site morbidity [11].

Comparatively, free or pedicled flap reconstruction, although theoretically offering more robust coverage, would have entailed significantly higher operative complexity and risk in this patient, given advanced atherosclerotic disease and a history of contralateral transfemoral amputation [4]. Emerging alternatives, such as dermal regeneration templates or bioengineered skin substitutes, may represent useful adjuncts in selected cases; however, they remain costly, often require prolonged integration time, and still depend on adequate perfusion and infection control [4]. In the present case, the simplicity, reproducibility, and adaptability of NPWT followed by split-thickness grafting offered a pragmatic and effective solution.

Several limitations of this approach must be acknowledged. Prolonged treatment duration and the need for multiple surgical interventions may not be feasible in all clinical settings. Additionally, long-term durability of grafted circumferential wounds in ischemic limbs remains a concern, necessitating careful follow-up and optimization of vascular risk factors in accordance with vascular surgery guidelines [2,10]. Nevertheless, the absence of recurrent infection and sustained graft take in this case supports the viability of this strategy when applied judiciously.

This case aligns with an emerging body of literature suggesting that limb salvage may be achievable even in extensive ischemic-infective defects traditionally considered indications for amputation [4,10]. While conclusions cannot be generalized from a single case, the present report contributes valuable clinical insight by illustrating a reproducible, staged treatment pathway that may be considered before resorting to major limb amputation in selected high-risk patients.

## Conclusions

This case demonstrates that even extensive circumferential skin and subcutaneous tissue loss of the lower leg secondary to bullous cellulitis complicating a chronic arterial ulcer can be successfully managed with a staged limb-salvage approach. Careful infection control, serial debridement, and negative pressure wound therapy allowed optimization of the wound bed and enabled definitive coverage with split-thickness skin grafting. In patients with advanced vascular disease and limited functional reserve, particularly those with prior contralateral major amputation, limb preservation is of paramount importance. A multidisciplinary, reconstructive strategy can prevent bilateral limb loss, preserve mobility, and maintain quality of life, even in high-risk elderly patients.
